# Regulation of T3SS synthesis, assembly and secretion in *Pseudomonas aeruginosa*

**DOI:** 10.1007/s00203-022-03068-5

**Published:** 2022-07-10

**Authors:** Hend Selim, Tharwat E. E. Radwan, Amany M. Reyad

**Affiliations:** Botany Department, Faculty of Science, Fayoum University, Fayoum, Egypt

**Keywords:** *Pseudomonas aeruginosa*, T3SS, Fluorescence microscopy, Cyclic di-GMP

## Abstract

T3SS is an important virulence factor of *Pseudomonas aeruginosa* and has a central role in the infection process. However, the functional regulation of the T3SS by environmental signals is poorly understood. In our lab, we use fluorescence microscopy to study protein kinetics in real-time in live cells. In *P. aeruginosa,* results have shown that T3SS appears as bright foci at the cell membrane with no specific arrangement. In addition, T3SS is tightly controlled as it appears under a limited time period with the highest intensity at 3 h then disappears. Surprisingly, only 2.5% of the all assembled T3SS in the population have detectable ExoS synthesis. While T3SS assembly and ExoS synthesis increased under high salt concentration, they unexpectedly were not affected by different cyclic di-GMP levels. On the other hand, T3SS itself has an effect on the cyclic di-GMP levels inside the cell. Data have shown that despite T3SS in *P. aeruginosa* and *Yersinia enterocolitica* belong to the same the group, the two systems differentiate greatly in activity and regulation. We can conclude that every T3SS is unique and thus further studies are needed to elucidate the functional regulation of each system to better help effective inhibitor design.

## Introduction

Many pathogenic gram-negative bacteria translocate effector proteins into host cells through a type III secretion system (T3SS) (Galán et al. 1999). It is crucial for virulence in many important human pathogens such as *Shigella*, *Salmonella*, and pathogenic *Escherichia coli*, taking millions of lives each year (Coburn et al. 2007). In *Pseudomonas aeruginosa*, T3SS is associated with high antibiotic resistance and elevated mortality rates in animals (Sawa et al. 2014). Moreover, strains with active T3SS are persistent and increase disease severity in humans; this reveals its key role in nosocomial infections (Hauser 2009; Sawa et al. 2014; Anantharajah et al. 2016).

While the translocated effector proteins are species-specific, the injectisome itself is conserved. This makes it an attractive target for the development of broadly applicable anti-virulence therapeutics (Charro et al. 2015). However, currently there are no efficient inhibitors with broad application (Charro et al. 2015; Gu et al. 2015) and our limited knowledge of T3SS molecular function and regulation prevents the rational design of such compounds.

**Fig. 1:**
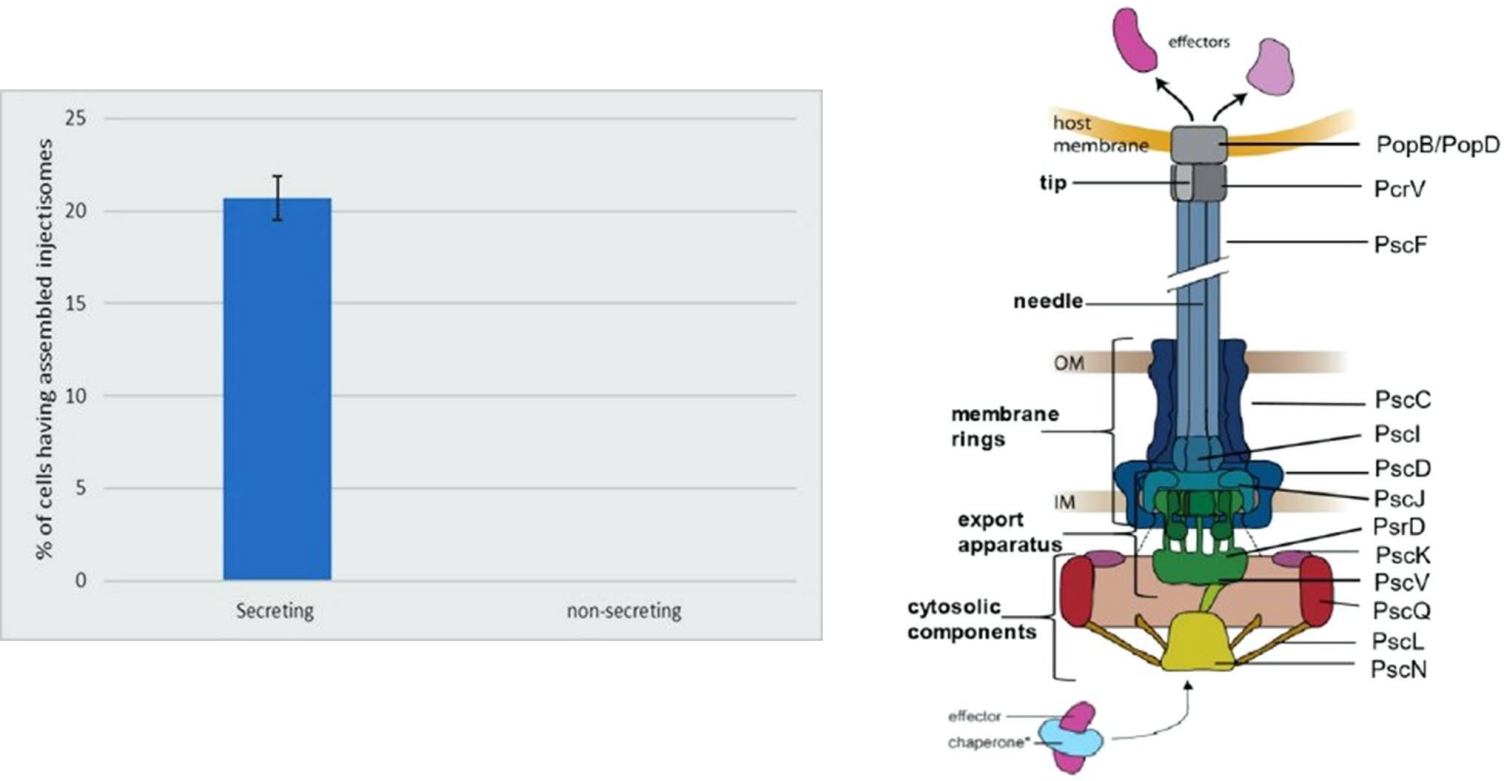
(Left) The assembly of the T3SS under secreting and non-secreting condition in *P. aeruginosa.* (right) Schematic overview of the T3SS. The main known injectisome components are labeled after the Psc nomenclature which is specified for the T3SS of Pseudomonas. *OM* outer membrane, *IM* inner membrane. Modified from Diepold and Wagner 2014

T3SSs are complex systems that consist of 20 proteins, forming a macromolecular complex that spans the bacterial inner membrane, outer membrane, and the host cell membrane (Nguyen et al. 2000). T3SSs include inner structures, inside the bacterial cell membrane, which consist of a cytosolic ATPase, a cytoplasmic ring, an inner membrane export apparatus, a basal body, which goes in the bacterial inner and outer membrane, and finally a translocation pore (Büttner 2012). In addition, outer structures which are represented by a needle base with the needle tip reaching into the environment and can form—through secreted translocator proteins—a translocon into the host cell (Fig. 1). Recently, in-situ structures of the T3SS, including the cytosolic complex, have been described (Hu et al. 2017). But more work is required to understand the functional regulation of the inner parts of the system.

In *P. aeruginosa* and other bacteria with T3SS, the cytosolic complex consists of four conserved proteins that are crucial to the function of the T3SS. Those proteins include an ATPase, PscN; its negative regulator, PscL; PscQ, and protein (PscK) (Bange et al. 2010). They are essential for many T3SS functions including the selection and preparation of substrates for export and transduction of energy (Spaeth et al. 2009; Lara-Tejero et al. 2011, Diepold et al. 2015).

Recently, research groups have discovered that the cytosolic components work in a highly dynamic and adaptive network to control protein export by T3SS (Frank et al. 1991; Finck‐Barbançon et al. 1997, Zhang et al. 2017). In this study, PscQ cytosolic component was selected for the investigation of the T3SS in *P. aeruginosa*. Strain DL001 (GFP- PscQ) which has fluorescent fusion protein, GFP integrated at the *N*-terminus of PscQ into the genome was used. The cytosolic protein PscQ can easily be detected by live-cell fluorescence microscopy and localized at the cell membrane. Based on previous studies, PscQ foci are considered a fully assembled injectisome as its homolog YscQ (T3SS cytosolic protein *Yersinia enterocolitica*). It is worth mentioning that both *Y. enterocolitica* and *P. aeruginosa* belong to the same T3SS family (Büttner 2012).

The T3SS in *P. aeruginosa* is a tightly controlled and highly regulated system. Regulation of T3SS mainly proceeds at two levels: the injectisome formation which is a relatively slow process, and effector secretion which occurs very fast once it is induced (Hauser 2009). A highly complex regulatory network involves a partner-switching system, overlapping with it is cyclic AMP and cyclic di-GMP signaling systems, global regulators, two-component systems, and RNA binding proteins that regulate T3SS transcription and/or synthesis positively or negatively (Williams et al. 2019).

Among the factors that have an effect on the gene expression of T3SS are host cell contact and low Ca^2+^, DNA damage, metabolic stress, and osmolarity. High salt concentrations found extracellularly activate T3SS genes transcription with the maximum effect detected at 200 mM NaCl (Hornef et al. 2000; Rietsch and Mekalanos 2006). In addition, cyclic di-GMP is a very important signaling molecule and is involved in the lifestyle shift between the planktonic motile phase connected to the acute infection to the sessile phase associated with chronic infection (Tamayo et al. 2007; Jonas et al. 2009). C-di-GMP is synthesized by 3_,_5-diguanyl cyclase (DGC) protein from two GTP residues. Conversely, phosphodiesterase (PDE) proteins degrade c-di-GMP molecules into pGpG (5-phosphoguanylyl-(3–5)-guanosine) or two GMP molecules. The level of c-di-GMP inside the cell is determined by the antagonistic activities of the two proteins PDE and DGC (Petrova and Sauer 2011; Valentini and Filloux 2016). In *P. aeruginosa*, the RetS/GacS signaling cascade and c-di-GMP are exploited in the conversation between acute infections which include cytotoxicity and motility through the T3SS, and chronic infections associated with the formation of biofilm and T6SS (Goodman et al. 2009).

Based on these findings, we aim to analyze the T3SS in *P. aeruginosa* under controlled conditions, to understand the assembly, activity, localization and distribution of the system in live cells and how regulation is controlled by some external environmental factors. This information can help in designing broadly applicable inhibitors for the T3SS.

## Materials and methods

### Plasmid construction

Plasmid construction was carried out using Phusion polymerase (New England Biolabs). Mutators for exchange of genes on the *P. aeruginosa* genome were created as previously described (Diepold et al. 2011). Inserted sequences were confirmed by sequencing (Eurofin Genomics). *P. aeruginosa* mutants were generated by allelic exchange, resulting in the exchange of wild-type gene sequences with the mutated gene (Kaniga et al. 1991a, b). *E. coli* Top10 was used for cloning and it was grown on Luria–Bertani (LB) agar plates or in LB medium at 37 °C (Table 1).

**Table 1:** Bacterial strains and constructs: used in this study are listed

Strain	Relevant characteristics	Reference
PAO1	Wild type of *P. aeruginosa*	Sophie Bleves
DL001	PAO1GFP-PscQ Mutator plasmid: pDL001(pKNG101:preFP-pscQ) Linker between gfp and PscQ	Lampaki 2018
DL003	PAO1 ΔPscV Mutator plasmid: pDL003(pKNG101* ΔpscV)(Containing N-terminal and C-terminal sequence of pscV)	Lampaki 2018

Plasmid	Relevant characteristics	
pJN105	Expression vector	New England Biolabs
pHS002	pJN105-dgcA,	This study
pAD731	pJN105- exoS-mCherry-ssrA	This study
pAD732	pJN105-PA2133-FLAG**	This study

*pKNG101:oriR6K sacBR oriTRK2 strA (suicide vector) (Kaniga et al. 1991a, b)

**FLAG: A short polypeptide marker useful for recombinant protein identification (Hopp et al. 1988)

### pHS002

In order to construct pHS002 (pJN105-dgcA). Forward and reverse primers AD1023 and AD1024 (Table 2) were used to amplify the diguanylate cyclase enzyme gene from plasmid template pAD482 (pBAD dgcA). The resulting insert was digested with EcoRI and XbaI and ligated into pJN105 cut with the same restriction enzymes. As pBAD is not a suitable expression vector for *P. aeruginosa*.

**Table 2:** Primer sequences are listed

Name	Sequence
AD1023	GACTGAATTCATGAAAATCTCAGGCGCCCG
AD1024	GACTTCTAGATCAAGCGCTCCTGCGCTTG
AD1202	TATATCTAGAGGCAGCCATTAGAGCAGT
AD1203	CTTGCTCACCGATTGAATATGCATGATGGTTGC
AD1204	TGCATATTCAATCGGTGAGCAAGGGCGAGGAG
AD1205	TATAGAATTCGCtcgaGTTAGGCTGCTAGA
AD1206	TATAGAATTCATGAACGGTTCCCCACAGG
AD1207	TATATCTAGA*TCA*CTTATCATCGTCGTCCTTGTAGTCACCTCCTTGTCTGCTCGCCAGCGCCTCGA

### pAD731

For the construction of the T3SS expression reporter plasmid with genotype pJN105: exoS::mCherry-ssrA. Forward and reverse primers AD1202, AD1203 (Table 2) were used to amplify the start of the ExoS T3SS effector gene from the *P. aeruginosa* genome. While, primers AD1204, AD1205 forward, and the reverse was used to amplify mCherry-SsrA from template pAD708 (pBAD- mCherry-SsrA). In a second amplification round the DNA mixture of purified first-round PCRs were amplified using external primers AD1202 and AD1205 (Table 2) to give overlapping insert of exoS::mCherry-ssrA. The resulting insert was digested with EcoRI and XbaI and ligated into pJN105 cut with the same restriction enzymes.

### pAD732

For construction of the pAD732 (pJN105:PA2133-FLAG). Primers AD1206 and AD1207 forward and reverse were used to amplify the phosphodiesterase enzyme gene (PA2133) from the *P. aeruginosa* genome. The resulting insert was digested with EcoRI and XbaI and ligated into pJN105 cut with the same restriction enzymes.

### Measuring T3SS assembly and ExoS synthesis secreting and non-secreting conditions

For T3SS assembly determination, strain DL001 culture was inoculated to 0.15 OD and incubated under secreting ((LB + 20 mM MgCl_2_ + 200 mM NaCl_2_ + 5 mM EGTA) and non-secreting (LB + 20 mM MgCl_2_ + 200 mM NaCl_2_ + 5 mM CaCl_2_) medium (Frank 1997) for 3 h at a temperature of 37 °C shaking at 180 rpm. For time course experiment, OD_600_ was measured at specific time-points, total cell samples were taken and 1.5 μl of the sample was analyzed under fluorescence microscopy. The strains functionality (effector secretion) was tested using mass spectrometry and compared to WT. All strains were functioning normally.

For measuring ExoS synthesis, a T3SS reporter plasmid pAD731 was designed. The idea is that when T3SS is active and effectors are being translated and expressed, we can detect fluorescence under the fluorescence microscope at the single cell level. Strains (DL001/pAD731) were incubated under secreting conditions for 3 h at a temperature of 37 °C and analyzed under fluorescence microscopy.

### Fluorescence microscopy

For analysis with fluorescence microscopy, agar slides were prepared using depression slides. For agar pads, 1.5% of agarose (low gelling temperature, SIGMA, Germany) was added to 2 ml minimal medium (HEPES, (NH_4_) SO_4_, NaCl, Sodium glutamate, K_2_SO_4_, Casamino acids) and heated at 75 °C until agarose melted. Then 80 μl was spotted on a depression slide (Marienfeld GmbH & Co.KG, Germany. For microscopy, Deltavision Spectris Optical Sectioning Microscope (Applied Precision, Issaquah, USA) was used equipped with an UPlanSApo × 100/1.40 oil objective (Olympus, Tokyo, Japan) and 1.6 auxiliary magnification. To take differential interference contrast (DIC) and fluorescence photomicrographs, an Evolve EMCCD Camera (Photometrics, Tucson, USA) was used. To visualize GFP fluorescence signals, GFP filter sets (Ex 490/20 nm, Em 525/30 nm) were used together with 500 ms exposure time. While mCherry filter sets were used to visualize mCherry fluorescence signals per every image, a Z-stack containing 11 frames per wavelength with a spacing of 150 nm was acquired. After taking photomicrographs, stacks were de-convoluted using the software softWoRx v.6.5.2 with moderate settings (Applied Precision, USA). After this, the de-convoluted picture was further analyzed with ImageJ software. For this, the stack in the DIC frame where the bacterium is entirely in focus was selected for analyzing the cells and the corresponding fluorescence. FIJI-ImageJ software was used to analyze all images. The brightness and contrast of every image was adjusted to remove the background.

### Measuring T3SS assembly and ExoS synthesis under different conditions

To investigate how the level of c-di-GMP affects the T3SS system at the molecular levels in terms of assembly and secretion, two plasmids were constructed, namely pAD732 which encodes for phosphodiesterase enzyme that degrades c-di-GMP and pHS002 which encodes for diguanylate cyclase enzyme that synthesizes c-di-GMP and transformed into strain DL001 and WT. After 3 h of incubation at 37 °C in secreting medium supplemented with gentamycin and induction of the plasmids using 0.5% arabinose, cell aggregation was quantified by taking 2 ml of the cultures and letting it sit for 1 h. and measuring the optical density (OD_600_) before and after vigorously suspending cells. A high ratio means less aggregation of cells, while a low ratio means higher cell aggregation associated with biofilm formation.

To study the effect of different levels of c-di-GMP on the T3SS assembly, GFP-PscQ Foci were analyzed by fluorescent microscopy after 3 h of incubation in secreting medium containing GM and arabinose for Constructs DL001/pJN105, DL001/pAD732, DL001/pHS002. To investigate the effect of c-di-GMP on the function of T3SS, effector secretion was quantified using label-free mass spectrometry techniques (Lampaki 2020).

Additionally, to investigate if T3SS activity has any effect on c-di- GMP levels, cell aggregation was evaluated. In addition, the optical density optical of cultures under different conditions of T3SS active and non-active was measured. OD_600_ was measured at first then cultures were left to settle down for 1 h. Then, OD_600_ was measured again. The percentage of cell fraction in suspension was calculated by dividing ODs before and suspension. Three constructs were made with strain DL002 (no T3SS activity) namely, DL002 + pJN105, DL002 + pAD732, and DL002 + pHS002. Then OD_600_ was measured after 3 h of incubation in secreting and non-secreting conditions and analyzed under fluorescence microscopy.

To study the effect of salt concentration of NaCl on growth, T3SS assembly, and ExoS synthesis, two LB media with low salt concentration (3 g/L) and high salt concentration (10 g/L) were used. To measure NaCl salt concentration on growth, culture of strain (DL001) was inoculated to the OD_600_ of 0.15 and incubated shaking for 6 h and OD_600_ was recorded every 2 h. In addition, a culture of strain (DL001/pAD731) was inoculated to the OD_600_ of 0.15 and incubated shaking for 3 h at 37°C. Then, samples were imaged under fluorescence microscopy using a GFP filter for T3SS assembly and mCherry filter for ExoS synthesis.

### Statistical analysis

Data were statistically analyzed using a one-way analysis of variance (ANOVA test) using SPSS Statistical Package Program version 23. Mean of the treatments were compared by Duncan multiple range test when the differences were significant. Level of significance in all tests was *P* ≤ 0.05. The results are expressed as means ± standard error (SE).

## Results and discussion

### Characterization of the *P. aeruginosa* T3SS

In this study, we investigated T3SS function and regulation by studying assembly and ExoS synthesis under different conditions using mainly live cell fluorescence microscopy. Using fluorescently-tagged protein, this technique enables the study of the in vivo localization of the T3SS components on a protein level. To date, Rietsch and Mekalanos (2006) were the first ones to visualize the T3SS of *P. aeruginosa* using immuno-labeled needle protein PscF. Thus, a more precise approach was needed to investigate the localization and kinetics of a single or multiple proteins of T3SS in real time in live cells.

Results revealed that PscQ protein are successfully expressed forming distinctive bright foci that are localized at the cell membrane and have no specific pattern or spatial arrangement. PscQ foci are considered a fully assembled injectisome as its homolog YscQ (T3SS cytosolic protein *Yersinia enterocolitica*). Foci were only detected at secreting medium as low calcium concentration is needed to induce secretion, while high calcium concentration represses secretion (Frank 1997) (Fig. 1). T3SS gene transcription is activated, as a consequence of activation of T3SS secretion induced by environmental signals such as low calcium concentration (Dasgupta et al. 2004).

**Fig. 2:**
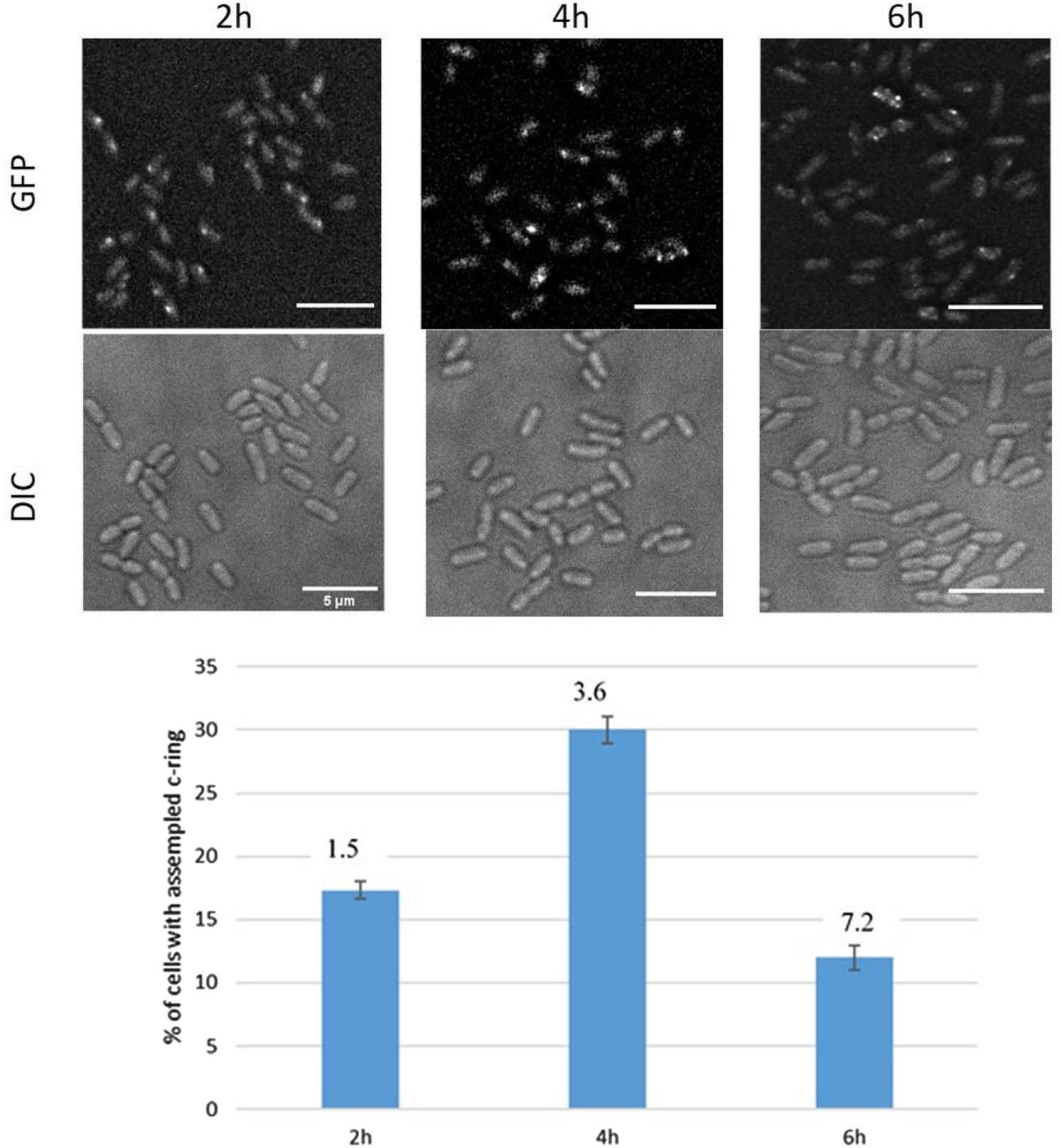
(up) Assembly of the T3SS over time under a fluorescence microscope. Samples were taken every 2 h after incubation of Strain DL001 in secreting medium. (down) Assembly of the T3SS over time under the fluorescence microscope. cells containing foci were counted and divided by the total cell number. OD measurements are indicated above each column

**Fig. 3:**
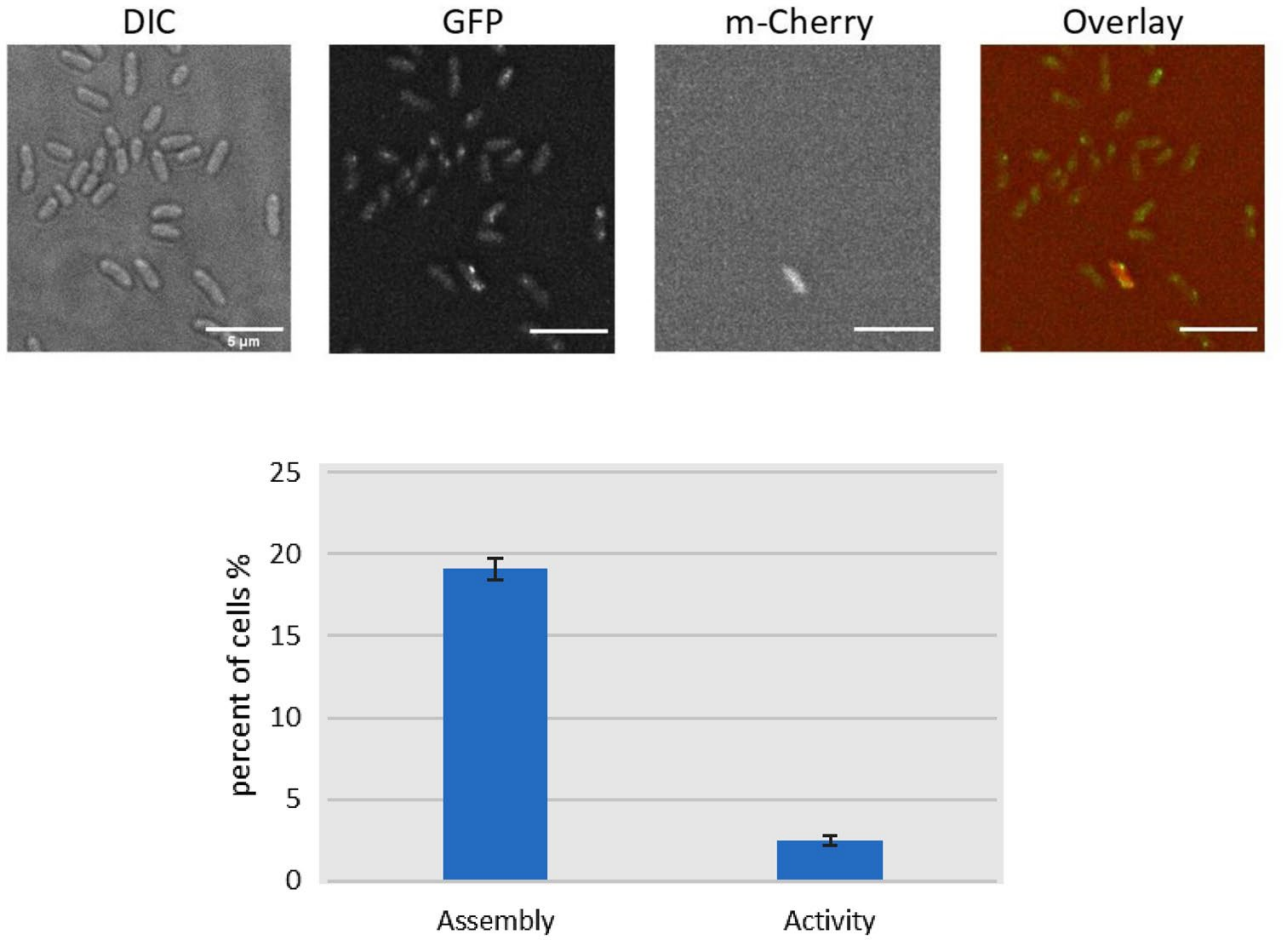
(up) Images of fluorescence microscopy showing ExoS-mCherry synthesis of T3SS of *P. aeruginosa* using reporter plasmid pAD731. Strains were incubated in secreting medium for 3 h at 37 °C. (down). ExoS-mCherry synthesis and PscQ foci of T3SS of *P. aeruginosa*. The ExoS-mCherry synthesis was evaluated using T3SS reporter plasmid in strain DL001/pAD731

Time course microscopy results revealed that *P. aeruginosa* has a heterogeneous population which means that not all cells show foci for T3SS. In addition, the number of positive cells containing T3SS foci starts at a very low number at an earlier time point (2 h) then increases gradually until it reaches the peak at 3–4 h, then decreases gradually with the increase of cell density at 6 h (Fig.  2). This can be explained that there may be a relationship between T3SS assembly and the cell density of the population and consequently the quorum sensing system. With respect to ExoS synthesis, a T3SS reporter plasmid (pAD731) was designed. In this plasmid, the promoter of ExoS effector (secreted by T3SS) was added, in addition to mCherry fluorescent protein and a degradation tag at the end (ssrA) (Farrell et al. 2005) which added to prevent the unspecific accumulation of the signal with time. The idea is that when T3SS is active and effectors are being expressed, we can detect fluorescence under the microscope. Results showed that very few signals for ExoS-mCherry fluorescence were detectable in the *P. aeruginosa* population. Only 13% of the cells with positive T3SS signals (assembled injectisomes) have a detectable signal for ExoS fluorescence, which means that only 2.5% of the population have ExoS synthesis (Fig. 3). An explanation might be that T3SS injectisomes in *P. aeruginosa* are very active even with a very small number and their activity is only needed for a specifically limited stage of the infection cycle. Another explanation is that the T3SS activity is compensated by the other large complex network of virulence factors of *P. aeruginosa* including but not limited to T2SS, T6SS, flagellum, toxins, and biofilm formation.

### Measuring T3SS assembly and ExoS synthesis under different conditions

#### Salt concentration

**Fig. 4:**
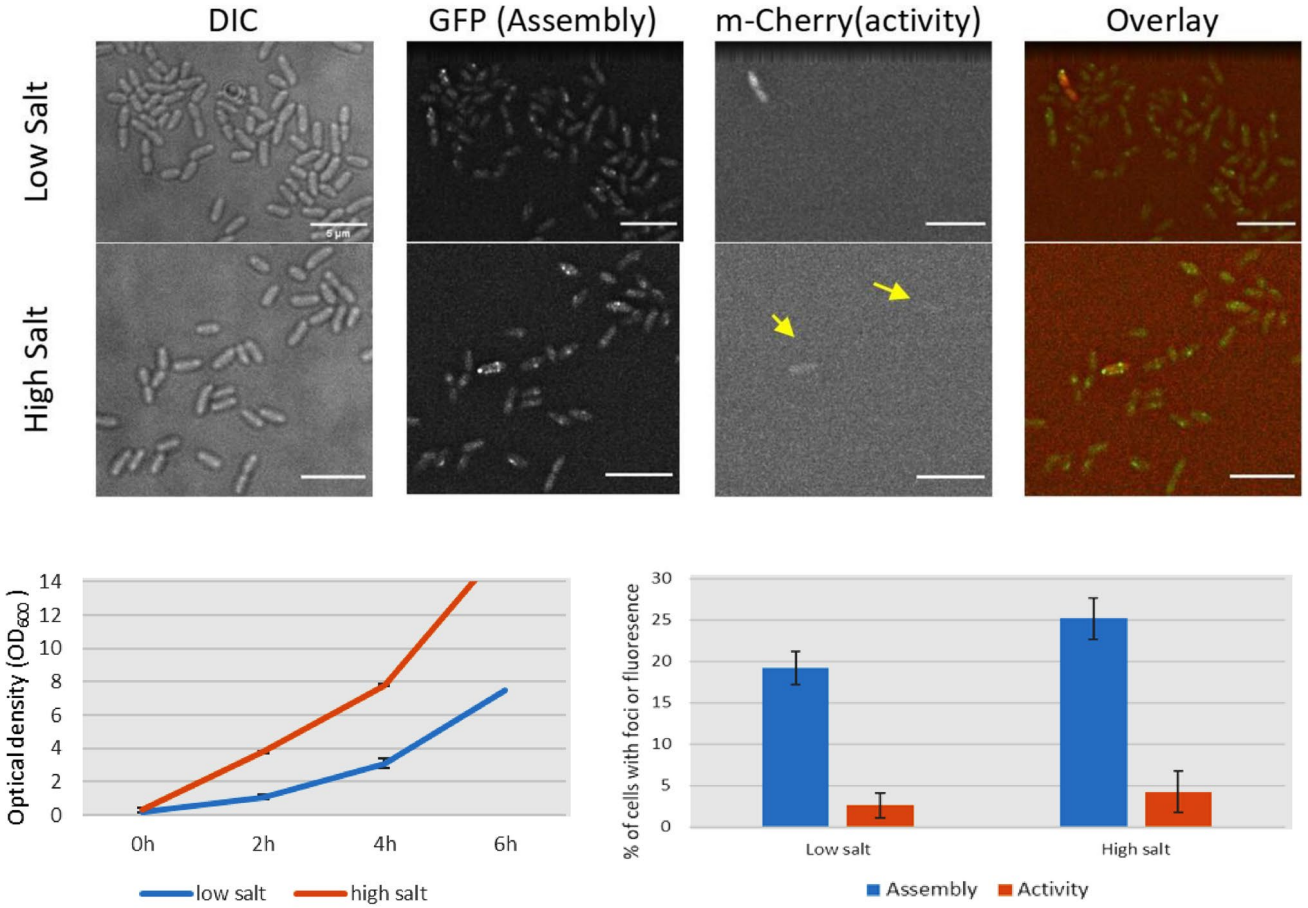
(up) Effect of NaCl salt concentration on the PscQ foci and ExoS-mCherry synthesis of T3SS of *P. aeruginosa*. Strain (DL001 + pAD731) was imaged under fluorescence microscopy, GFP filter was used to study the PscQ foci and m-Cherry filter was used to study the ExoS-mCherry synthesis of T3SS. (down) (right) Effect of NaCl salt concentration on ExoS-mCherry synthesis and PscQ foci of T3SS of *P. aeruginosa*. Strain (DL001/pAD731) was imaged under fluorescence microscopy using an m-Cherry filter and GFP filter, cells with fluorescence were quantified. (left) Effect of NaCl salt concentration on the growth rate of *P. aeruginosa* strain DL001. Optical density (OD_600_) was recorded every 2 h for a 6 h period

From the data in Fig. 4, we can observe that high salt concentration increased the rate of growth of *P. aeruginosa* strains. Furthermore, it increased T3SS assembly ExoS synthesis. In consistence with that, researchers have showed that hyper‐osmotic stress conditions were found to significantly induce T3SS genes analyzed by Microarray techniques (Aspedon et al. 2006). Osmolarity has been considered the reason for this salt effect, as the same effect can be induced using other solutes such as sucrose (Aspedon et al. 2006; Rietsch and Mekalanos 2006). They also suggested that there is a possible link between the cAMP signaling pathway, osmotic pressure and the regulation of T3SS, as levels of intracellular cAMP increased with increasing salt concentration medium (Yahr and Wolfgang 2006; Rietsch and Mekalanos 2006).

#### Cyclic di-GMP

**Fig. 5:**
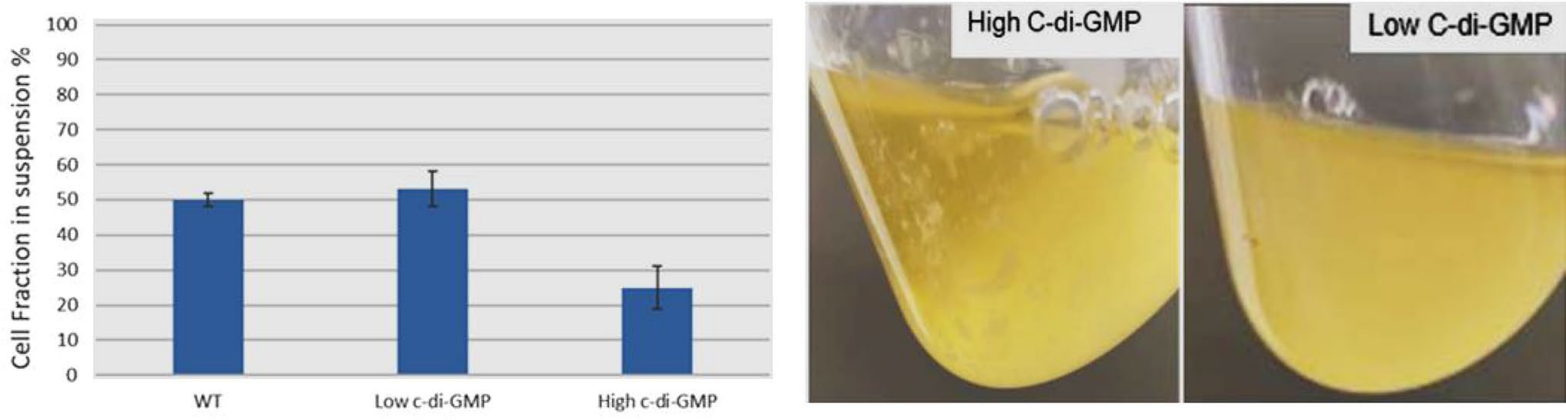
(right) Effect of different levels of cyclic di-GMP on cell aggregation. (left) Effect of different levels of cyclic di-GMP on the cell aggregation, where we can see aggregates of cells under high cyclic di-GMP which are not found under low cyclic di-GMP

From the data in Fig. 5, we can observe a cell aggregation in pHS002 containing construct but not in the ones with pAD732 or the controls and we take that as a readout that our constructs are functional and we were able to change c-di-GMP levels inside the cell.

**Fig. 6:**
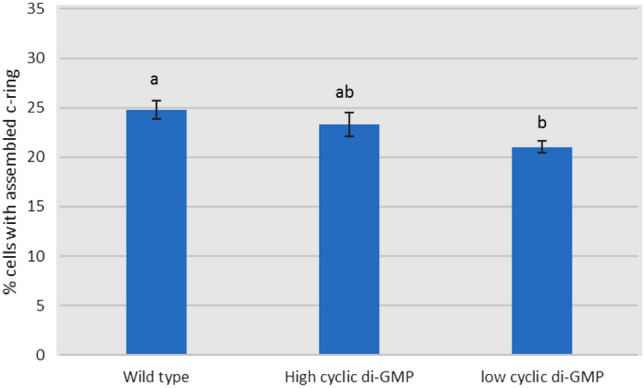
Effect of different levels of cyclic di-GMP on assembly of T3SS injectisomes. **a**, **b**, **c** Average in having different superscripts are differ significantly (*P* ≤ 0.05)

The study of the effect of different levels of c-di-GMP on the T3SS assembly indicates that there is no big difference in the number of assembled injectisomes under low and high c-di-GMP compared to the control (Fig. 6). Inconsistent with these results, some studies have reported that high levels of c-di-GMP cause repression in the T3SS gene expression (Moscoso et al. 2011). However, other studies showed that both T3SS and T6SS are active at the beginning of biofilm formation (Mikkelsen et al. 2011).

**Fig. 7:**
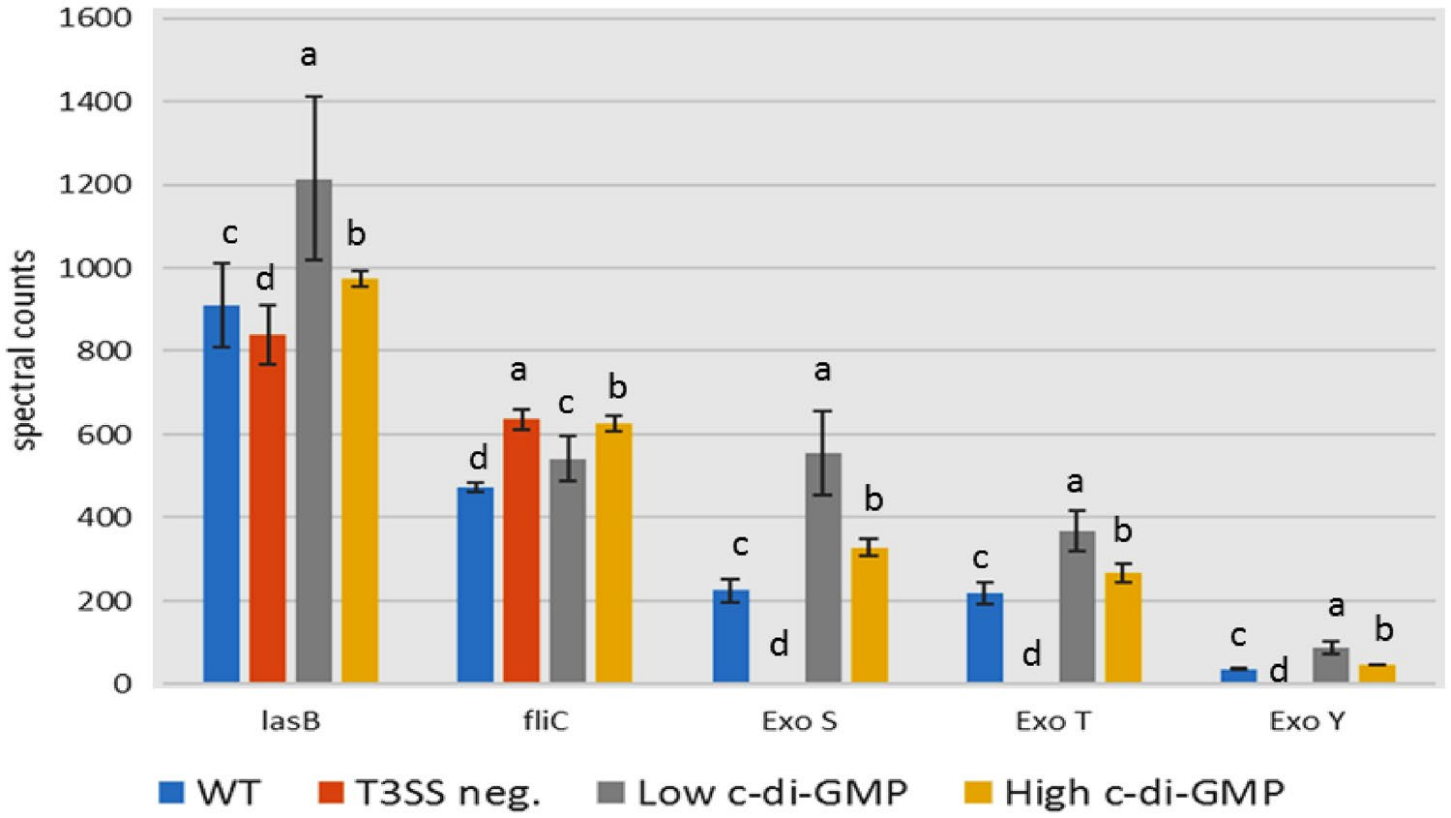
Effect of different levels of cyclic di-GMP on activity (effector secretion) of T3SS injectisomes. Secreted T3SS proteins (Exo S, Exo T, Exo Y) were evaluated in the supernatant using mass spectrometry. In addition to non-T3SS secreted proteins (Las B, FliC) as a control. **a**, **b**, **c** Average in having different superscripts are differ significantly (*P* ≤ 0.05)

To investigate the effect of c-di-GMP on the function of T3SS, results showed that the effectors' secretion (Exo S, T, Y) is different under variable conditions when compared to background proteins (LasB, FliC) that are secreted independently of T3SS. Strain DL002 (T3SS negative) has no T3SS effectors secretion. As we can see, low c-di-GMP activates T3SS secretion when compared to the WT. On the other hand, there is almost no difference between high c-di-GMP and the WT. The overall conclusion from these experiments is that high or low levels of c-di-GMP do not affect T3SS assembly or activity. In contrast, T3SS activity and high c-di-GMP affect cell aggregation and they are independent of each other (Fig. 7).

**Fig. 8:**
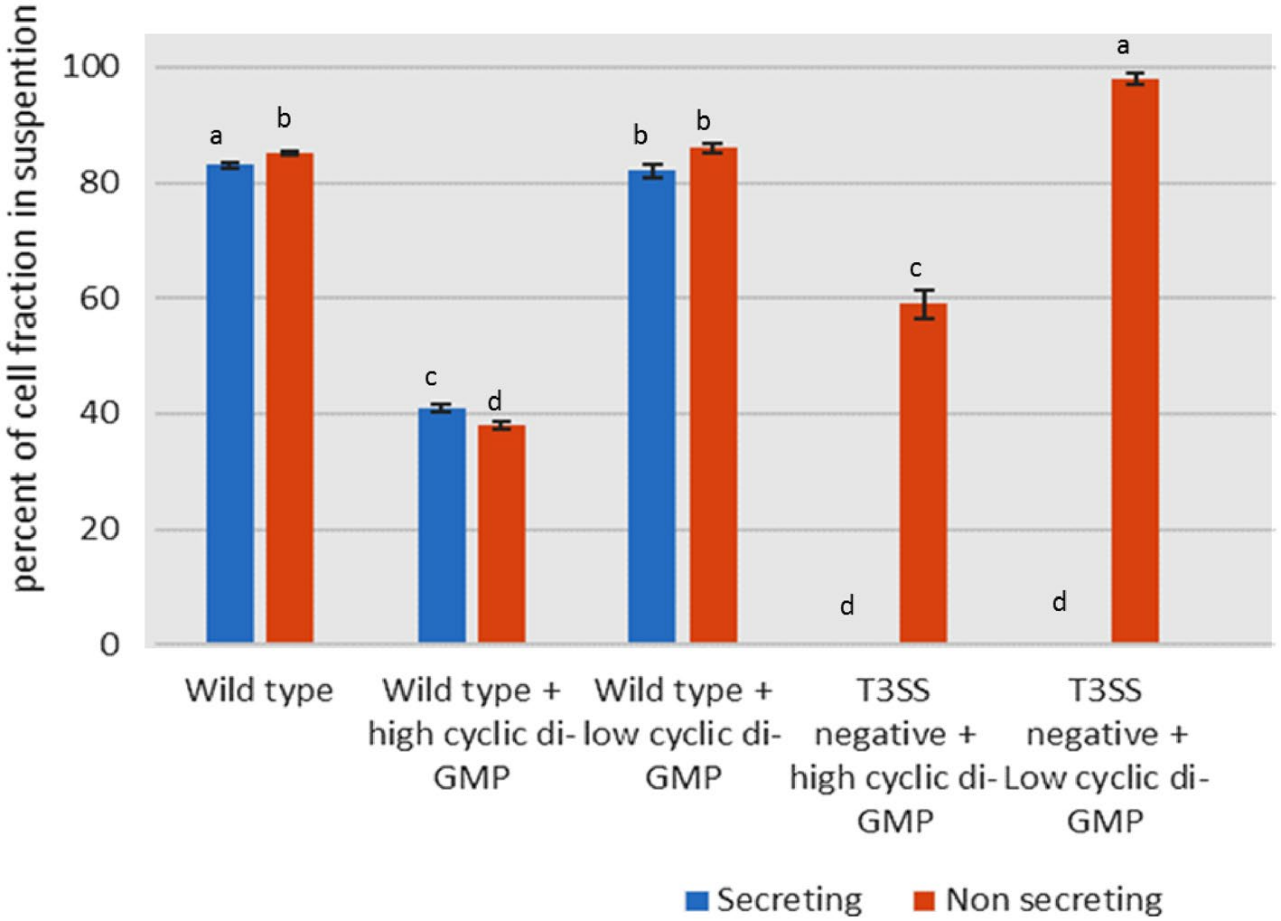
Effect of T3SS activity on levels of cyclic di-GMP. Evaluated by cell aggregation using optical density, the percent of cell fraction in suspension was calculated by dividing ODs before and suspension. **a**, **b**, **c** Average in having different superscripts are differ significantly (*P* ≤ 0.05)

Additionally, in order to investigate if T3SS activity has any effect on c-di- GMP levels. Results indicate that T3SS increases cell aggregation by presumably increasing the c-di-GMP level inside the cell. This can be confirmed by the increase of the percent of cell fraction in suspension in T3SS negative strain under high and low c-di-GMP levels. While, there is no big difference between secreting and non- secreting conditions in all cases (Fig.  8). An explanation is that there is a relationship between T3SS as shown by many researches such as Moscoso et al. 2011 that proved that the conversion between T3SS and T6SS can be induced by artificially manipulating c-di-GMP levels inside the cell. Another possibility is that effect of T3SS gene induction on C-di-GMP could be indirect and the pscV mutation could affect genetic regulation. The overall conclusion from these experiments is that high or low levels of c-di-GMP do not affect T3SS assembly or activity. In contrast, T3SS activity and high c-di-GMP affect cell aggregation and they are independent of each other.

Although *P. aeruginosa* and *Y. enterocolitica* (another pathogen studied in our lab) are phylogenetically different and have different lifestyles (Dong et al. 2018). Yet, they both belong to the Ysc family of T3SS. Comparing different regulatory pathways will enable us to determine if the respective regulatory pathway is specific for *P. aeruginosa* or conserved and to define common and species-specific pathways.

In our lab, we can observe from the results that there are many differences in the assembly and activity of the T3SS in *Y. enterocolitica* and *P. aeruginosa*. In Y. enterocolitica, 99% of cells have assembled EGFP- YscQ with a uniform arrangement that is present under secreting and non-secreting conditions (Milne-Davies et al. 2019). Also, in *P. aeruginosa*, only 25% of cells have assembled EGFP-PscQ with scattered positions and foci are only present under secreting and disappear under non-secreting conditions. Concerning T3SS activity, results showed that in *P. aeruginosa* only 2.5% of the population have ExoS synthesis. As for *Y. enterocolitica*, almost all cells are active and secreting (Milne-Davies et al. 2019).

### Conclusion

A general and collective conclusion that we can extract from our work is that every T3SS is unique and has a different complex regulation mechanism, even if it exists in related species such as in *P. aeruginosa and Y. enterocolitica*. So, the inhibitor's design should be specific to each T3SS. More experiments are needed to understand more about the different regulatory elements and how they respond to external environmental signals. In addition, it could help understand the relationship between different virulence factors. *P. aeruginosa* is instrumental to investigate the high virulence and adaptability of the bacterium.
